# Silencing long non-coding RNA Kcnq1ot1 alleviates pyroptosis and fibrosis in diabetic cardiomyopathy

**DOI:** 10.1038/s41419-018-1029-4

**Published:** 2018-09-24

**Authors:** Fan Yang, Ying Qin, Jie Lv, Yueqiu Wang, Hui Che, Xi Chen, Yanan Jiang, Anqi Li, Xi Sun, Er Yue, Long Ren, Yang Li, Yunlong Bai, Lihong Wang

**Affiliations:** 1 Department of Endocrinology, The Second Affiliated Hospital of Harbin Medical University, Harbin, China; 2 Translational Medicine Research and Cooperation Center of Northern China, Heilongjiang Academy of Medical Sciences, Harbin, China; 3 Department of Pharmacology (State-Province Key Laboratories of iomedicine-Pharmaceutics of China, Key Laboratory of Cardiovascular Medicine Research, Ministry of Education), College of Pharmacy, Harbin Medical University, Harbin, China

## Abstract

Diabetes cardiomyopathy (DCM) is a critical complication of long-term chronic diabetes mellitus and is characterized by myocardial fibrosis and myocardial hypertrophy. It has been suggested that DCM is related to pyroptosis, a programmed cell death associated with inflammation. The long non-coding RNA Kcnq1ot1 is involved in different pathophysiological mechanisms of multiple diseases, including acute myocardial damage and arrhythmia. Our previous study found that Kcnq1ot1 was elevated in left ventricular tissue of diabetic mice. However, whether Kcnq1ot1 is capable of regulating pyroptosis and fibrosis in high glucose-treated cardiac fibroblasts remains unknown. The aim of the study was to investigate the mechanisms of Kcnq1ot1 in DCM. Our study revealed that silencing Kcnq1ot1 by a lentivirus-shRNA improved cardiac function and fibrosis, ameliorated pyroptosis, and inhibited TGF-β1/smads pathway in C57BL/6 mice. In vitro, experiments revealed that Kcnq1ot1 and pyroptosis were activated in cardiac fibroblasts treated with 30 mmol/l glucose. Furthermore, Kcnq1ot1 knockdown by a small interfering RNA decreased caspase-1 expression. Bioinformatic prediction and luciferase assays showed that Kcnq1ot1 functioned as a competing endogenous RNA to regulate the expression of caspase-1 by sponging miR-214-3p. In addition, silencing Kcnq1ot1 promoted gasdermin D cleavage and the secretion of IL-1β, thus repressing the TGF-β1/smads pathway in high glucose-treated cardiac fibroblasts through miR-214-3p and caspase-1. Therefore, Kcnq1ot1/miR-214-3p/caspase-1/TGF-β1 signal pathway presents a new mechanism of DCM progression and could potentially be a novel therapeutic target.

## Introduction

Diabetic cardiomyopathy (DCM) is a vital complication of diabetes mellitus (DM) and is characterized by structural and functional dysfunction in the absence of hypertension, coronary artery disease, and other cardiac pathologies^1^. It has been suggested that cell death, mitochondrial dysfunction, and inflammation are all hallmarks of cardiac hypertrophy and myocardial fibrosis. The fibrosis is the characteristic pathological manifestation of DCM and enhances the risk of heart failure, arrhythmia and sudden death^2^. Therefore, the study of the mechanisms underlying myocardial fibrosis is essential.

Pyroptosis is a type of programmed cell death related to the activation of inflammation^3^. It has been reported that hyperglycemia activates nucleotide-binding oligomerization domain-like receptor pyrin domain containing (NLRP) 3 inflammasome, thus promoting pro-caspase-1 to caspase-1. The cleaved caspase-1 is then capable of converting the pro-interleukin-1β (pro-IL-1β) and pro-interleukin-18 (pro-IL-18) into matured IL-1β/IL-18^4^. Recently, studies have revealed that gasdermin D (GSDMD) is another critical component of the inflammasome and can be cleaved by activated caspase-1^5,6^. The cleaved *N*-terminal of GSDMD (GSDMD-N) is essential in the process of pyroptosis because it can promote the secretion of matured IL-1β and damage the plasma membrane^6^. More importantly, to our knowledge, the activation of the inflammasome and the release of cytokines can promote the deposition of collagens and fibrotic formation, further exacerbating the severity of DCM^7^. Previous studies have shown that the inhibition of pyroptosis by microRNA-9 (miR-9), miR-30d, NLRP3 gene silencing, and rosuvastatin etc. can improve the cardiac function of diabetic mice^8–11^. However, a comprehensive study focused on the molecular mechanisms underlying pyroptosis in cardiac fibroblasts and its effect on cardiac fibrosis is still lacking.

Long non-coding RNAs (lncRNAs) are a group of nonprotein coding RNA transcripts of length > 200 nucleotides^12^. LncRNAs regulate gene expression in epigenetic, transcriptional, and post-transcriptional levels. LncRNAs have been found to play critical roles in a variety of biological processes, such as cell proliferation and migration, inflammation, apoptosis, and autophagy^13^. The KCNQ1 opposite strand/antisense transcript 1 (Kcnq1ot1) is a lncRNA located in human chromosome 11p15.5^14^. A growing number of studies have shown that Kcnq1ot1 is involved in various diseases, including acute myocardial damage and arrhythmia^15,16^. Jin et al.^17 ^showed that Kcnq1ot1 could regulate caspase-1 expression by targeting miR-214-3p to promote cataract formation. However, the role and molecular regulatory mechanism of lncRNA Kcnq1ot1 in high glucose (HG)-induced pyroptosis and fibrosis is still unknown. Thus, the present study aimed to probe the ability of Kcnq1ot1 to modulate pyroptosis and cardiac fibrosis in the process of DCM.

In this study, we report for the first time that the lncRNA Kcnq1ot1 was significantly upregulated in diabetic myocardial tissues and HG-treated cardiac fibroblasts. Further investigations demonstrated that Kcnq1ot1 could act as a competitive endogenous RNA (ceRNA) for miR-214-3p to regulate the expression of caspase-1. Knockdown of Kcnq1ot1 ameliorated pyroptosis and fibrosis in vivo and in vitro.

## Results

### Silencing Kcnq1ot1 ameliorates cardiac function and fibrosis in diabetic mice

In the pre-experiment, three different siRNAs against Kcnq1ot1 were used to assess the efficiency of Kcnq1ot1 knockdown in cardiac fibroblasts. The results showed that the expression levels of Kcnq1ot1 were significantly decreased after transfection with all three siRNAs (Supplementary Figure 1a). The si-Kcnq1ot1-1 with the better efficiency at silencing Kcnq1ot1 expression was selected for the subsequent experiments. The lentivirus carrying shRNA targeting Kcnq1ot1 was constructed based on the sequence of si-Kcnq1ot1-1.

**Fig. 1:**
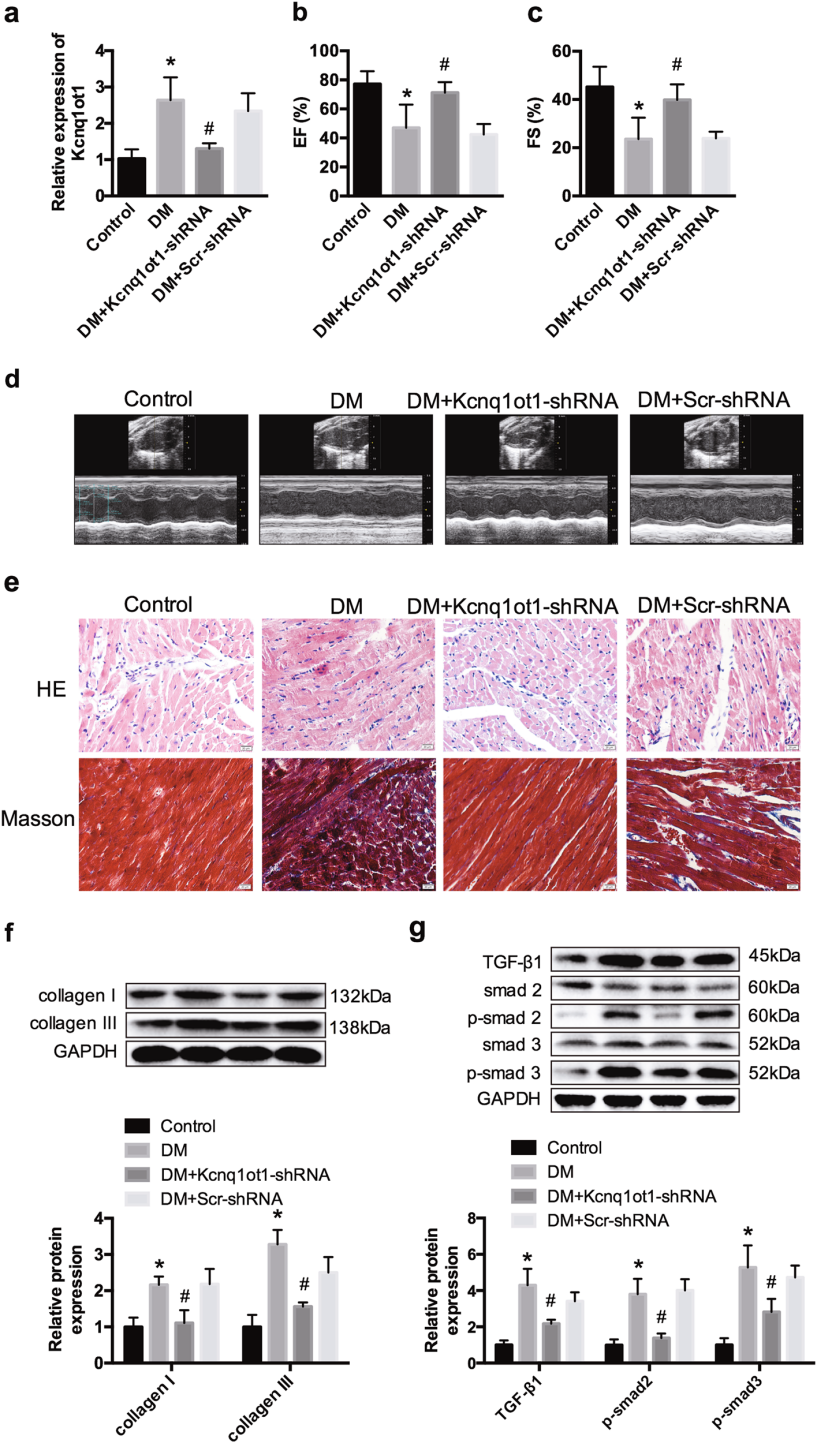
Silencing Kcnq1ot1 attenuates cardiac function and structure in diabetic mice **a** The expression of Kcnq1ot1 in left ventricle was detected by qRT-PCR. M-mode echocardiograms of left ventricle **b**, ejection fraction (EF) **c** and shortening fraction (FS) **d** are shown. **e** Hematoxylin-eosin (HE) and Masson’s trichrome staining were performed. Scale bar, 50 μm. **f** The expression levels of collagen I and collagen III were detected by western blot. **g** Relative protein expression of TGF-β1, p-smad2, and p-smad3 were detected by western blot. *P < 0.05 compared with the control group, ^#^*P* < 0.05 compared with the DM + Scr-shRNA group. *n* = 5 in each group

To investigate the potential function of Kcnq1ot1 in DCM, we established a streptozotocin (STZ)-induced diabetic model in C57BL/6 mice. As shown in Fig. 1a, Kcnq1ot1 expression was dramatically elevated in cardiac tissue of the diabetic mice and downregulated after injection with the Kcnq1ot1-shRNA lentivirus. Functionally, the echocardiographic data indicated a deterioration of the systolic and diastolic functions of the left ventricular, and the ejection fraction (EF) and fractional shortening (FS) were decreased in the DM group. However, these changes were restored to some extent after treatment with Kcnq1ot1-shRNA (Fig. 1b–d). Morphologically, hematoxylin and eosin stain (HE), and Masson’s trichrome staining revealed that the increased myocardial mass and collagen deposition induced in the diabetic heart were ameliorated after silencing Kcnq1ot1 expression (Fig. 1e). Furthermore, western blot analysis showed that collagen I and collagen III were significantly reduced after silencing Kcnq1ot1 (Fig. 1f). TGF-β1, phosphorylated smad2 (p-smad2) and p-smad3 expression levels were remarkably increased in the DM group; however, TGF-β1/smads signaling pathway was significantly inhibited in the DM + Kcnq1ot1-shRNA group (Fig. 1g).

### Kcnq1ot1 is involved in the regulation of pyroptosis in vivo

**Fig. 2:**
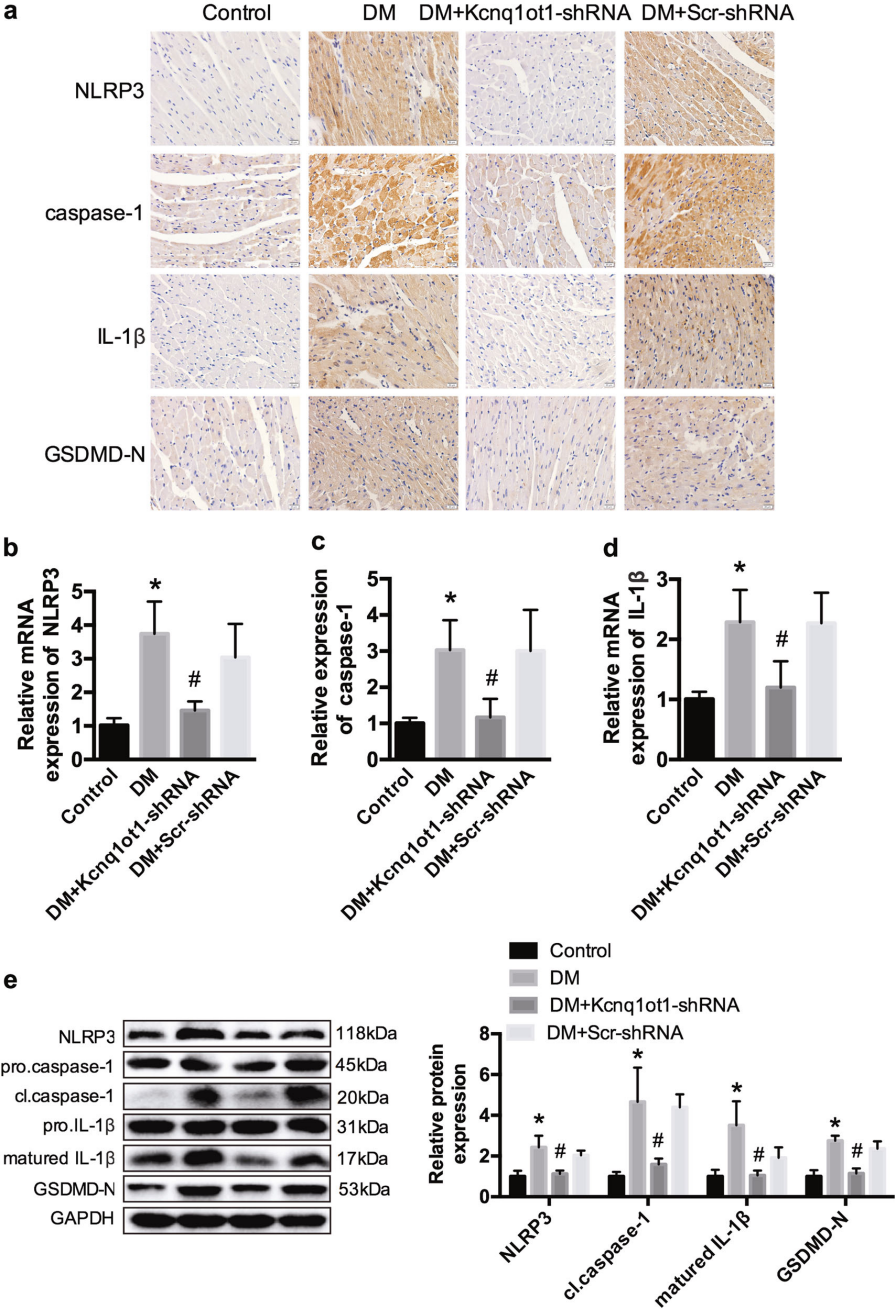
Kcnq1ot1 is involved in the regulation of pyroptosis in vivo **a** Immunohistochemistry analysis was performed to detect the expression NLRP3, caspase-1, IL-1β, and GSDMD-N. Scale bar, 20 μm. **b**–**d** qRT-PCR was conducted to analyze the mRNA expression of NLRP3, caspase-1 and IL-1β. **e** Western blot was conducted to determine the protein expression of NLRP3, caspase-1 and IL-1β. *P < 0.05 compared with the control group, ^#^*P* < 0.05 compared with the DM + Scr-shRNA group. *n* = 5 in each group

Immunohistochemistry analysis indicated that NLRP3, caspase-1, IL-1β, and GSDMD-N levels were significantly elevated in cardiac tissue of diabetic mice. However, the levels were reduced after Kcnq1ot1 silencing (Fig. 2a). The mRNA and protein expression levels of NLRP3, caspase-1, and IL-1β were remarkably increased in the DM group and were reduced after treatment with Kcnq1ot1-shRNA (Fig. 2b–e). Moreover, protein expression levels of GSDMD-N were significantly increased in the diabetic mice, and were reversed following Kcnq1ot1 inhibition (Fig. 2e). These data demonstrate that lncRNA Kcnq1ot1 modulates pyroptosis in the diabetic heart.

### Kcnq1ot1 and pyroptosis are activated in HG-treated cardiac fibroblasts

**Fig. 3:**
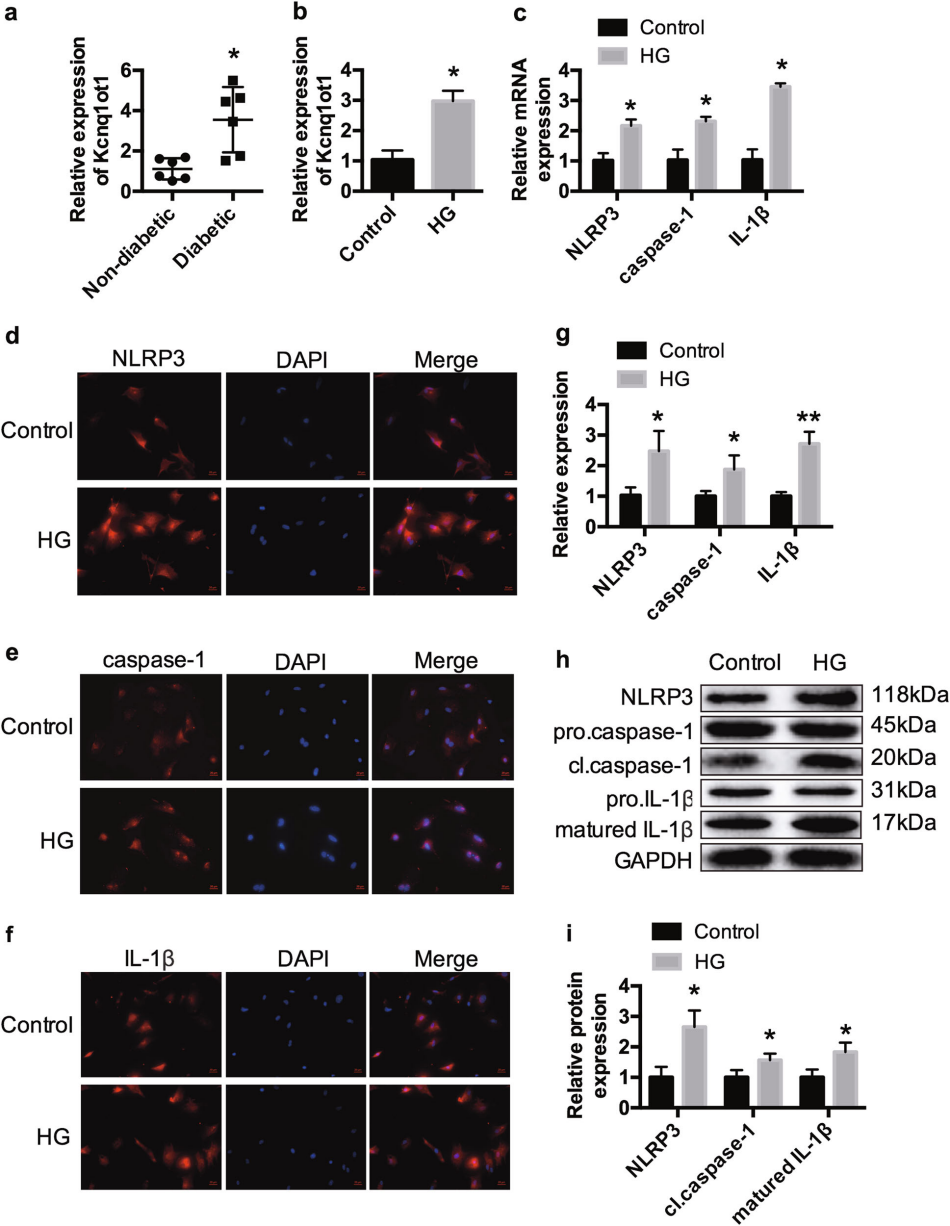
Kcnq1ot1 and pytoptosis are activated in HG-treated cardiac fibroblasts qRT-PCR was performed to measure the Kcnq1ot1 expression level in the serums of non-diabetic and diabetic patients **a**. *P < 0.05 compared with the non-diabetic group. *n* = 6 in each group. Cardiac fibroblasts of neonatal C57BL/6 mice were incubated with 5.5 mmol/L glucose (Control) or 30 mmol/L (high glucose, HG) for 24 h. The expression levels of Kcnq1ot1 were detected by qRT-PCR **b**. The expression levels of NLRP3, caspase-1 and IL-1β were determined by qRT-PCR **c**, immunofluorescence **d**–**g** and western blot **h**, **i**. *P < 0.05 compared with the control group. *n* = 3 in each group

We further confirmed the effects of Kcnq1ot1 on pyroptosis with in vitro experiments. Cardiac fibroblasts of neonatal C57BL/6 mice were incubated with either 5.5 mmol/L glucose (Control) or 30 mmol/L (HG) for 24 h. qRT-PCR revealed that Kcnq1ot1 was significantly elevated both in the serum of diabetic patients and HG-treated cardiac fibroblasts (Fig. 3a, b). Consistent with the in vivo results, the expression levels of NLRP3, caspase-1, and IL-1β were also elevated in the HG-treated cardiac fibroblasts as revealed by qRT-PCR, immunofluorescence, and western blot (Fig. 3c–i).

### miR-214-3p contains both Kcnq1ot1 and caspase-1-binding sites

**Fig. 4:**
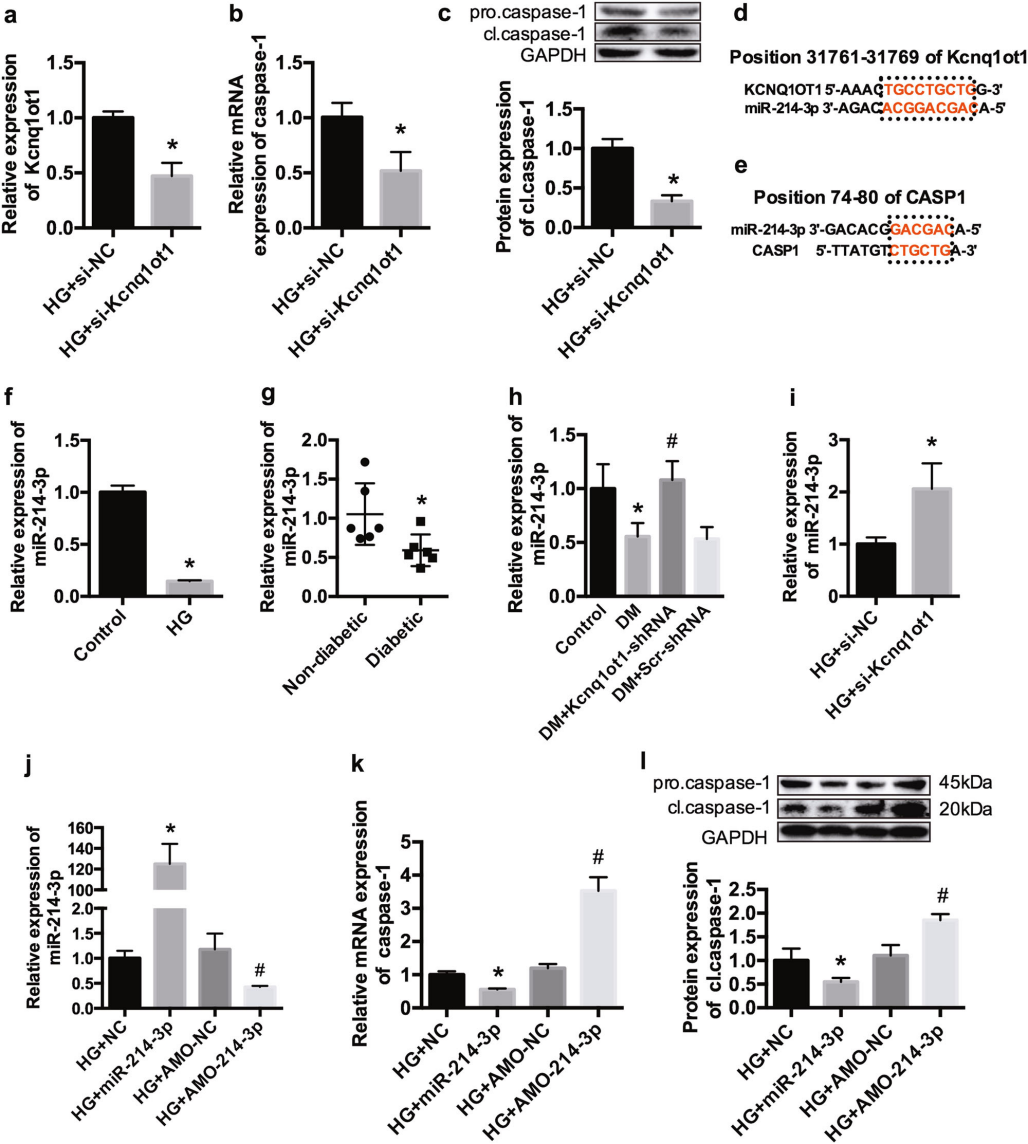
miR-214-3p has both Kcnq1ot1 and caspase-1-binding sites HG-treated cardiac fibroblasts were transfected with siRNA against Kcnq1ot1 and the negative control. The Kcnq1ot1 expression was detected by qRT-PCR **a**. qRT-PCR **b** and western blot **c** were performed to determine the mRNA and protein expression levels of caspase-1. Bioinformatic prediction revealed that miR-214-3p contained potential binding sites for both Kcnq1ot1 **d** and caspase-1 **e**. The relative expression of miR-214-3p was analyzed in cardiac fibroblasts **f**, serums of patients **g**, and myocardium of mice **h**. **i** miR-214-3p expression after transfection with si-Kcnq1ot1. Fibroblasts were transfected with miR-214-3p mimics or miR-214-3p inhibitor, and the expressions of miR-214-3p and caspase-1 were determined **j**–**l**. *P < 0.05 compared with the HG + NC group, ^#^*P* < 0.05 compared with the HG + AMO-NC group. *n* = 3 in each group

Silencing of Kcnq1ot1 by siRNA in the HG group (Fig. 4a) led to an evident decrease in the mRNA and protein expression levels of caspase-1 (Fig. 4b, c). In addition, the expression levels of caspase-1 were also downregulated after silencing Kcnq1ot1 with different siRNAs (Supplementary Figure 1b). We demonstrated that the results of the experiments were not due to the phenomenon of sequence specific off-target-derived phenotypes. Bioinformatic prediction analysis indicated that miR-214-3p potentially interacted with both Kcnq1ot1 and caspase-1. The binding sites are shown in Fig. 4d, e. Our team had previously demonstrated the complementary regulatory relationship between Kcnq1ot1 and miR-214-3p, as well as caspase-1 and miR-214-3p, by luciferase assays in HEK293T cells^17^. The binding sites of KCNQ1OT1 were cloned into the luciferase vector, and miR-214-3p mimics were co-transfected into HEK293T cells. The luciferase activity was significantly repressed when the cell was co-transfected with miR-214-3p mimics, whereas the mutation of the miR-214-3p target site reversed the repression (Supplementary Figure 2a and b). Moreover, a luciferase construction carrying the 3′-UTR of caspase-1 was generated. The results indicated that the translation of wild-type caspase-1 was inhibited by miR-214-3p mimics, as shown by the reduced luciferase signal, whereas the luciferase activity was unaffected by miR-214-3p mimics when the construction carried the 3′-UTR of caspase-1 with a mutant miR-214-binding site (Supplementary Figure 2c and d). qRT-PCR experiments indicated that miR-214-3p was decreased in the HG-treated cardiac fibroblasts (Fig. 4f), the serums of diabetic patients (Fig. 4g), and the cardiac tissue of diabetic mice (Fig. 4h). Silencing of Kcnq1ot1 resulted in an increased miR-214-3p expression in vivo and in vitro (Fig. 4h, i). We then performed gain-of-function and loss-of-function experiments to evaluate the effects of miR-214-3p on caspase-1 in fibroblasts. Fig. 4j indicates successful transfection. Overexpression of miR-214-3p led to decreased caspase-1 levels, whereas caspase-1 levels were increased after inhibition by miR-214-3p in the HG-treated cardiac fibroblasts (Fig. 4k, l).

### Silencing Kcnq1ot1 inhibits caspase-1 expression by targeting miR-214-3p in vitro

**Fig. 5:**
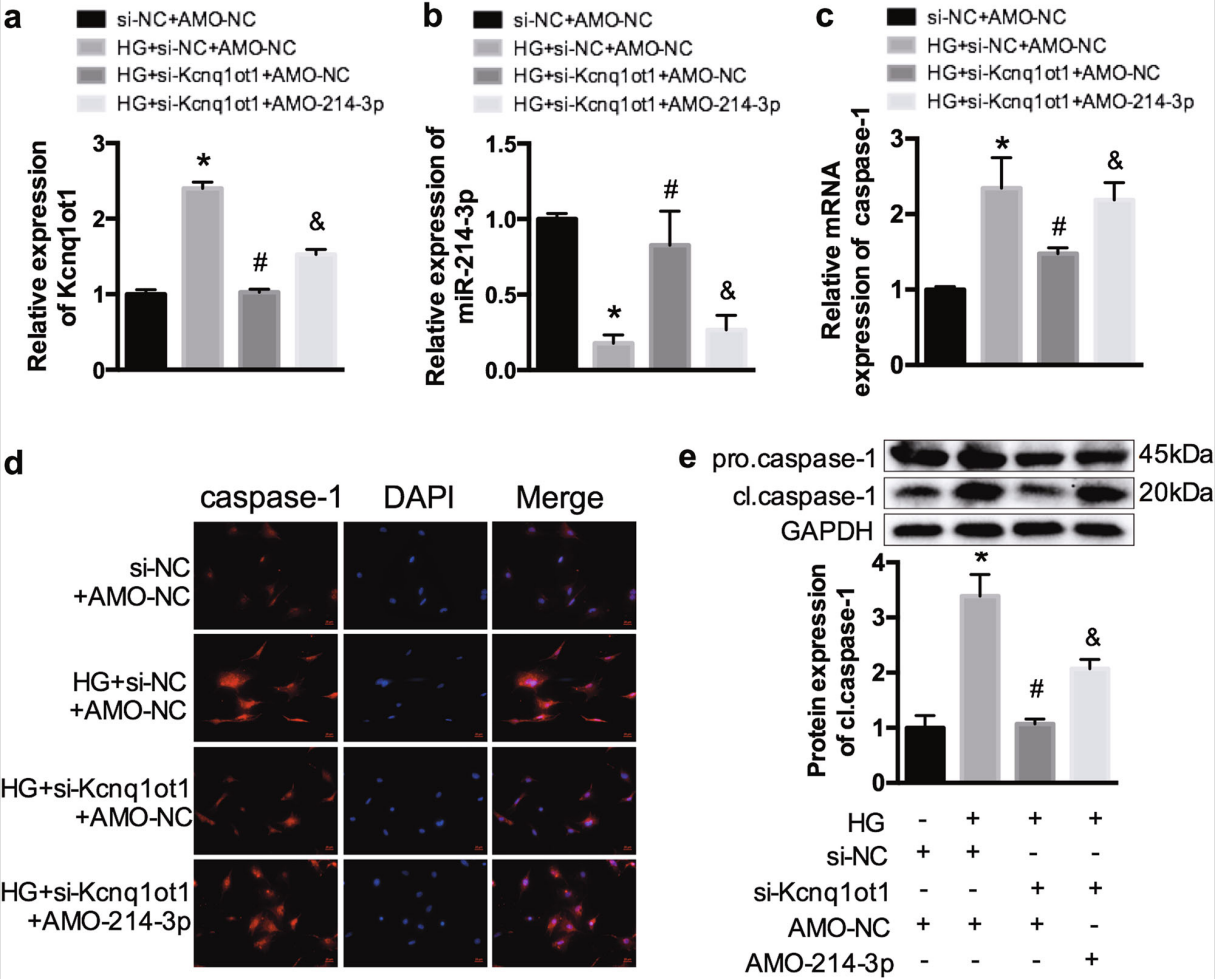
Kcnq1ot1 silencing alleviates caspase-1 via miR-214-3p **a** The HG-treated fibroblasts were transfected with si-Kcnq1ot1 with or without AMO-214-3p. The relative expression of Kcnq1ot1 was detected. **b** Relative miR-214-3p expression in each group. **c** The mRNA expression of caspase-1 in each group. **d** The caspase-1 expression was determined by immunofluorescence. **e** Western blot was conducted to detect the protein expression of cleaved caspase-1. *P < 0.05 compared with the si-NC + AMO-NC group, ^#^*P* < 0.05 compared with the HG + si-Kcnq1ot1 + AMO-NC group, ^&^*P* < 0.05 compared with the HG + si-Kcnq1ot1 + AMO-214-3p group. *n* = 3 in each group

In addition, cardiac fibroblasts incubated with 5.5 mmol/L glucose were transfected with si-NC and AMO-NC. Cells incubated with HG were divided into three transfection groups: those transfected with control si-NC and AMO-NC, si-Kcnq1ot1 and AMO-NC, and si-Kcnq1ot1 and AMO-214-3p. The expression levels of Kcnq1ot1 and miR-214-3p are shown in Fig. 5a, b. qRT-PCR indicated that the expression of caspase-1 was increased in the HG group and were significantly decreased after being transfected with si-Kcnq1ot1, whereas the inhibition was attenuated by co-transfection with AMO-214-3p (Fig. 5c). Simultaneously, immunofluorescence staining and western blot analysis also confirmed these results (Fig. 5d, e). Above all, we confirm that Kcnq1ot1 regulates caspase-1 expression by miR-214-3p in cardiac fibroblasts.

### Kcnq1ot1 regulates inflammation and pyroptosis of cardiac fibroblasts by sponging miR-214-3p

**Fig. 6:**
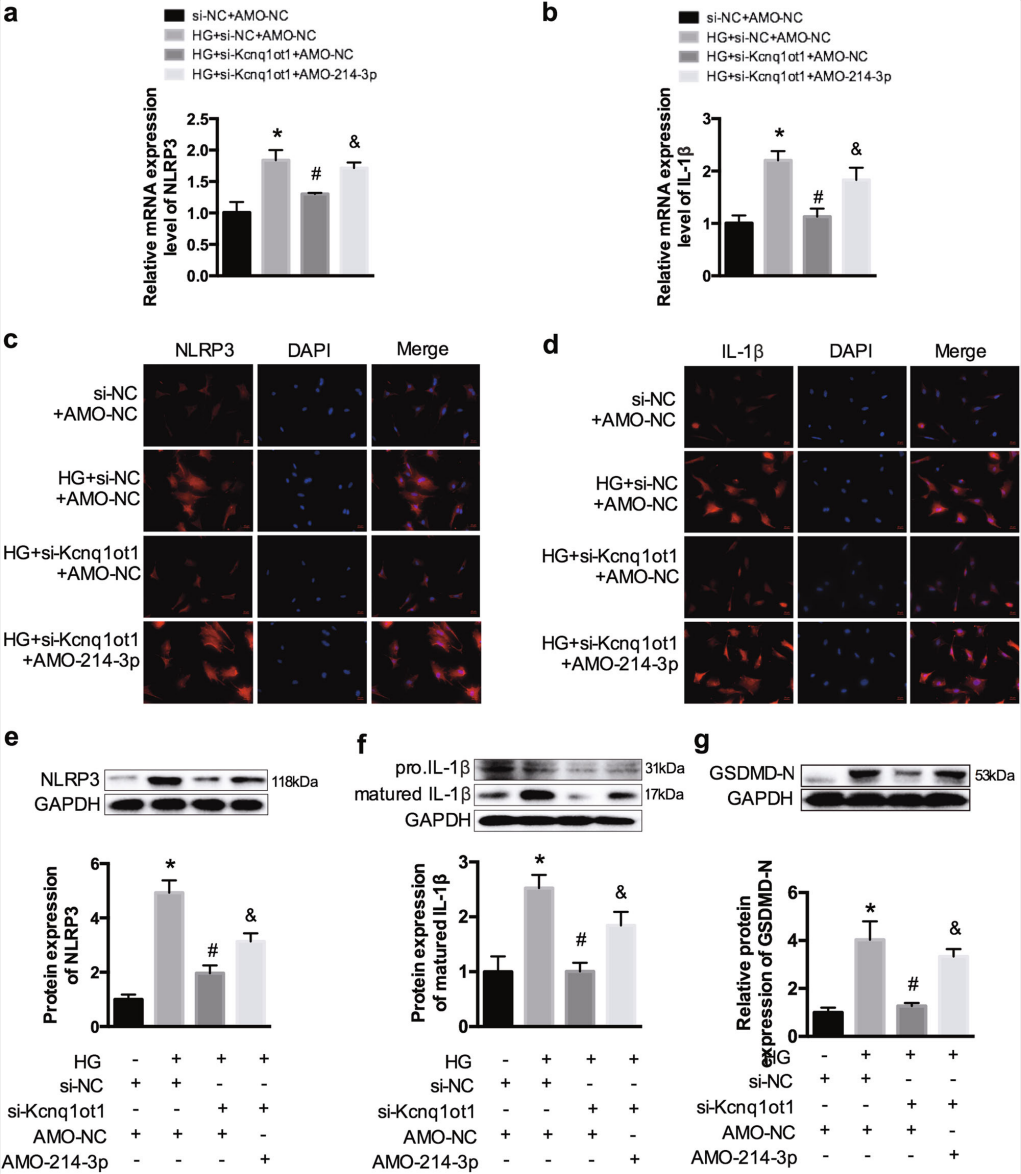
The texts overlap in figure 6g. So I want to replace it with a new figure 6 in the attachment. Kcnq1ot1/miR-214-3p pathway regulates inflammation in fibroblasts **a**, **b** qRT-PCR was conducted to detect expression of NLRP3 and IL-1β. **c**, **d** The expression levels of NLRP3 and IL-1β were determined by immunofluorescence. **e**–**g** Western blot was performed to determine the expression of NLRP3, IL-1β, and GSDMD-N. *P < 0.05 compared with the si-NC + AMO-NC group, ^#^*P* < 0.05 compared with the HG + si-Kcnq1ot1 + AMO-NC group, ^&^*P* < 0.05 compared with the HG + si-Kcnq1ot1 + AMO-214-3p group. *n* = 3 in each group

Cardiac fibroblasts were transfected with si-Kcnq1ot1 with or without AMO-214-3p in HG conditions. We observed that HG promoted increased NLRP3 and IL-1β expression, whereas these levels were decreased after silencing Kcnq1ot1. However, co-transfection with si-Kcnq1ot1 and AMO-214-3p promoted NLRP3 and IL-1β expression (Fig. 6a–f). In addition, western blot analysis was conducted to detect the expression of GSDMD-N. The results showed that GSDMD-N was evidently elevated in the HG-treated fibroblasts and was decreased after silencing Kcnq1ot1. However, the repressive effect was abrogated by AMO-214-3p (Fig. 6g). Therefore, silencing Kcnq1ot1 suppresses inflammation and pyroptosis by inhibiting the Kcnq1ot1/miR-214-3p pathway in HG-treated cardiac fibroblasts.

### Silencing Kcnq1ot1 alleviates fibrosis of HG-induced fibroblasts

**Fig. 7:**
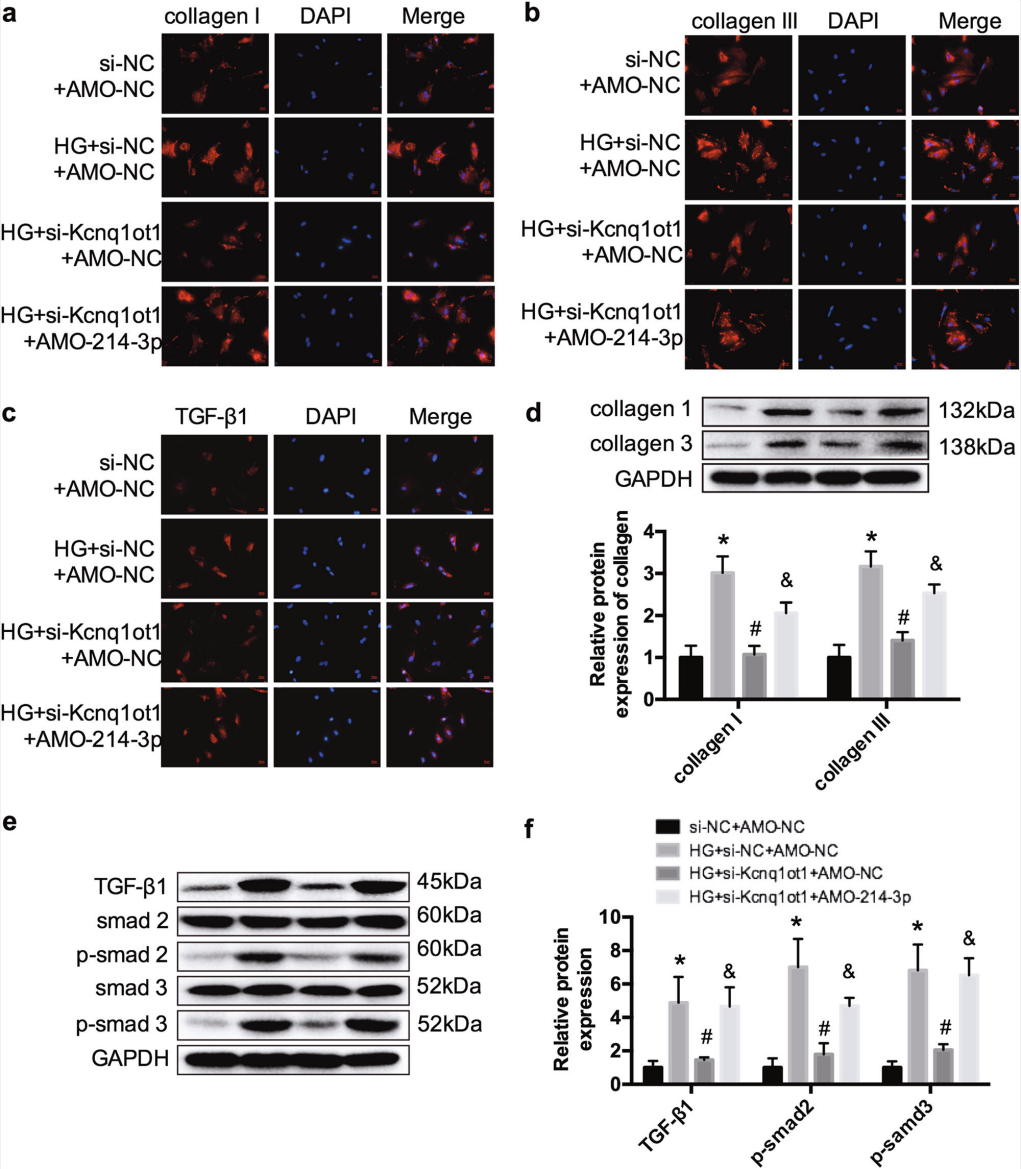
Kcnq1ot1 regulates fibrosis in HG-induced cardiac fibroblasts **a**–**c** Immunofluorescence was conducted to detect the expression levels of collagen I, collagen III, and TGF-β1. **d** The protein expression levels of collagen I and III were detected by western blot. **e**, **f** The protein expression levels of TGF-β1, p-smad2, and p-smad3 were determined by western blot. *P < 0.05 compared with the si-NC + AMO-NC group, ^#^*P* < 0.05 compared with the HG + si-Kcnq1ot1 + AMO-NC group, ^&^*P* < 0.05 compared with the HG + si-Kcnq1ot1 + AMO-214-3p group. *n* = 3 in each group

Previous studies revealed that HG could increase collagen I and collagen III expressions, and activate TGF-β1, p-smad2, and p-smad3. Here, we found that collagen I and collagen III expressions as well as the activated TGF-β1/smads pathway were repressed after silencing Kcnq1ot1, and were reversed by co-transfection with AMO-214-3p (Fig. 7). Therefore, we suggest that silencing Kcnq1ot1 alleviates inflammation and fibrosis of HG-induced fibroblasts through miR-214-3p.

## Discussion

DCM is an important cardiovascular complication of diabetes, but its pathogenesis is not yet fully elucidated. It has been documented that lncRNAs play vital roles in many diseases. The aim of the study was to investigate the biological function of the lncRNA Kcnq1ot1 in the development and procession of DCM. A critical finding of our research is that Kcnq1ot1 is overexpressed in DCM. Further, silencing Kcnq1ot1 alleviates myocardial dysfunction and attenuates myocardial fibrosis in STZ-induced C57BL/6 mice. In vitro studies revealed that silencing Kcnq1ot1 reduced caspase-1 expression by acting as an endogenous miR-214-3p sponge, thereby repressing downstream cytokines and leading to alleviation of HG-induced inflammation and fibrosis in cardiac fibroblasts. Thus, our study demonstrates the vital role of Kcnq1ot1 in DCM and establishes the lncRNA as a novel therapeutic target of DCM.

LncRNA Kcnq1ot1 has been shown to be closely related to a variety of diseases, including long QT syndrome, cataracts, cancers, and myocardial ischemia/reperfusion injury^14–18^. Recently, Gao et al.^14^ reported that Kcnq1ot1 induced macrophage polarization and improved osteolysis by inhibiting miR-21a-5p. In addition, Guo et al.^19^ found that Kcnq1ot1 promoted cell proliferation and metastasis in melanoma by acting as a ceRNA of miR-153. However, the relationship between Kcnq1ot1 and diabetes as well as its complications are rarely reported. Asahara et al.^20^ reported that Kcnq1ot1 was closely connected with pancreatic β-cell mass and was likely a key factor in the onset of diabetes. In the present study, our results reveal that lncRNA Kcnq1ot1 is highly expressed in myocardial tissues of STZ-induced diabetic mice and HG-treated cardiac fibroblasts. Inhibiting Kcnq1ot1 ameliorated caspase-1 expression significantly. We were the first to elucidate the differential expression of Kcnq1ot1 in DCM models.

LncRNAs are non-coding RNAs that can regulate the expression of protein-coding messenger RNAs. The ceRNA hypothesis is the most important mechanism of lncRNAs, positing that lncRNA can sponge miRNAs by competing binding sites and regulate gene expression^13,21,22^. The novel RNA cross-talk hypothesis provides a novel method of understanding the mechanisms of various disorders at the post-transcriptional level. In our study, using bioinformatic prediction, miR-214-3p was found to have both Kcnq1ot1 and caspase-1-binding sites, which was consistent with previous study in cataract^17^. Jin et al. have already demonstrated the complementary regulatory relationship among miR-214-3p, Kcnq1ot1 and caspase-1 using luciferase assay. Here, we performed functional assays and proved that Kcnq1ot1 can regulate the expression of caspase-1 by targeting miR-214-3p in HG-treated cardiac fibroblasts. Therefore, we suggest that Kcnq1ot1 might be an innovative therapeutic target for DCM through its regulation of caspase-1.

Pyroptosis is programmed cell death associated with inflammation and features pore formation, disruption on the plasma membrane, and cell swelling^3^. It was proven that pyroptosis participated in the process of DCM. Caspase-1 is vital to the regulation of pyroptosis and the maturation of cytokine^23^. In the present study, we reveal that Kcnq1ot1 can accelerate pyroptosis of HG-induced fibroblasts by enhancing caspase-1 expression in DCM. After silencing Kcnq1ot1, caspase-1 and its downstream inflammatory cytokines, IL-1β, were repressed remarkably via miR-214-3p targeting. It is worth emphasizing that previous studies have found that IL-1β, which is the central link in a variety of pathological processes, is closely related to cardiac fibrosis^24–26^. It was demonstrated that long exposure in IL-1β activated the TGF-β1 pathway and induced the transformation of microvascular endothelial cells to myofibroblasts, leading to increased collagen synthesis, promotion of myocardial remodeling and increased interstitial myocardial fibrosis^27–29^. Therefore, we have reason to speculate that silencing Kcnq1ot1 represses collagen deposition and alleviates myocardial fibrosis via inhibition of inflammatory factors. The ceRNA regulatory network, Kcnq1ot1/miR-214-3p/caspase-1/TGF-β1, plays an important role in the regulation of myocardial fibrosis in DCM.

In conclusion, we show that hyperglycemia induces higher expression of Kcnq1ot1 in cardiac fibroblasts. Downregulating Kcnq1ot1 alleviates pyroptosis and fibrosis via the miR-214-3p/caspase-1/TGF-β1 pathway. Our study is the first to demonstrate the expression and function of Kcnq1ot1 in DCM and put forward a new method of treatment for DCM based on a non-coding RNA.

## Materials and methods

### Serum samples of patients

The serums were extracted from healthy subjects or diabetic patients in the Second Affiliated Hospital of Harbin Medical University (*n* = 6 per group). Hypertension, coronary artery disease, and some other heart diseases were precluded in all patients. Patients involved in this research signed an informed consent. All experiments were approved by the ethical committee of Harbin Medical University.

### Animal model and treatment

Male C57BL/6 mice weighing 18–20 g were purchased from the animal experiment center of the Second Affiliated Hospital of Harbin Medical University. Diabetes were induced in the mice by intraperitoneal injection of 50 mg/kg/day STZ (Sigma, St. Louis, MO, USA) for 5 days. After 72 h, mice with blood glucose of > 16.7 mmol/L in the tail vein as measured by a Contour glucose meter (Roche, Germany) were considered successful diabetic models (DM)^30^. Diabetic mice were administered with either the Kcnq1ot1 lentivirus-shRNA (DM + Kcnq1ot1-shRNA) or scramble shRNA (DM + Scr-shRNA) (GenePharma, Shanghai, China). The sequence of the shRNA is GGTAGAATAGTTCTGTCTT. Then, 1 × 10^9^ TU lentivirus-shRNA was dissolved in 50 μL saline and injected into the tail vein of diabetic mice^31^. All mice were kept for 8 weeks and were anesthetized by avertin.

### Cell culture and transfection

The primary cardiac fibroblasts were extracted from hearts of one- to 3-day-old neonatal C57BL/6 mice. The isolation and culture methods were described previously^32^. The cells were cultured with different concentration of glucose: 5.5 mmol/L glucose (Control) and 30 mmol/L glucose (HG) for 24 h at 37 °C and 5% CO_2_ conditions. Cells were transfected with small interfering RNAs (siRNAs) against Kcnq1ot1 (si-Kcnq1ot1), miR-214-3p mimics (miR-214-3p), anti-miRNA oligonucleotides (AMO) of miR-214-3p (AMO-214-3p), or corresponding negative control (si-NC, NC, AMO-NC) designed and synthesized by RIOBIO (Guangzhou, China) for 48 h. The procedure was according to the manufacturer’s instructions. The sequences were as follows: si-Kcnq1ot1-1: GGTAGAATAGTTCTGTCTT; si-Kcnq1ot1-2: GCAGTTATTGAAACCTCTA; si-Kcnq1ot1-3: CCACATCACAGCAACCTAA; miR-214-3p mimics: forward, 5′-ACAGCAGGCACAGACAGGCAGU-3′, reverse, 3′-UGUCGUCCGUGUCUGUCCGUCA-5′; miR-214-3p inhibitor, 5′-mAmCmUmGmCmCmUmGmUmCmUmGmUmGmCmCmUmGmCmUmGmU-3′.

### Echocardiography

Two-dimensional M-mode echocardiography was detected by a Vevo1100 high-resolution imaging system (VisualSonics, Toronto, ON, Canada) to evaluate the cardiac function. EF and FS of the left ventricle were derived using the machine.

### HE and Masson’s trichrome staining

The cardiac tissues from the left ventricle fixed in 4% paraformaldehyde were embedded in paraffin and cut into 5 μm-thick sections. The sections were stained with HE and Masson’s trichrome separately. Morphology of the myocardium and deposition of collagen were observed by fluorescence microscope (IX71 Olympus, Japan).

### Total RNA isolation and quantitative real-time RT-PCR (qRT-PCR)

Total RNA in the human serums was extracted by Trizol LS (Invitrogen, USA), and the total RNA of cardiac fibroblasts and cardiac tissues was extracted by Trizol (Invitrogen, USA). RNAs were reversed-transcribed using the ReverTra Ace qPCR RT kit (Code No. FSQ-101, Toyobo, Japan). To detect the expression levels of miR-214-3p, we changed the Random Primer Mix to the miR-214-3p-specific RT-primers, which were designed by RIOBIO (Guangzhou, China), according to the manufacturer’s protocol and a previous study^33^. cDNA was amplified and were detected by an ABI 7500 fast system (Applied Biosystems, CA, USA). U6 was used as internal control for miR-214-3p and GAPDH for all others. The primers sequences are:

Kcnq1ot1 forward: 5′-GCACTCTGGGTCCTGTTCTC-3′,

Kcnq1ot1 reverse: 5′-CACTTCCCTGCCTCCTACAC-3′;

miR-214-3p forward: 5′-TATACATCAAACAGCAGGCACA-3′,

miR-214-3p reverse: 5′-CATTCGATCTTCTCCACAGTCTC-3′;

NLRP3 forward: 5′-GTGGAGATCCTAGGTTTCTCTG-3′,

NLRP3 reverse: 5′-CAGGATCTCATTCTCTTGGATC-3′;

caspase-1 forward: 5′-ACACGTCTTGCCCTCATTATCT-3′,

caspase-1 reverse: 5′-ATAACCTTGGGCTTGTCTTTCA-3′

IL-1β forward: 5′-CCCTGCAGCTGGAGAGTGTGG-3′

IL-1β reverse: 5′-TGTGCTCTGCTTGAGAGGTGCT-3′

GAPDH forward: 5′-ATCACTGCCACCCAGAAGAC-3′

GAPDH reverse: 5′-TTTCTAGACGGCAGGTCAGG-3′

U6 forward: 5′-CTCGCTTCGGCAGCACATATACT-3′

U6 reverse: 5′-ACGCTTCACGAATTTGCGTGTC-3′

### Protein extraction and western blot analysis

Total protein samples were extracted and run on 10% sodium dodecyl sulfate polyacrylamide gel electrophoresis. Proteins were then transferred to nitrocellulose membranes. After blocking with bovine serum albumin (BSA), the membranes were incubated with primary antibodies of NLRP3 (Boster Biological Technology, Wuhan, China, catalog BA3677, 1:800), caspase-1 (Cell Signaling Technology, Danvers, MA, USA, catalog 2225, 1:1000), IL-1β (Cell Signaling Technology, catalog 12703, 1:1000), GSDMD-N (Bioss, Beijing, China, catalog bs-14287R 1:1000), collagen I (Abcam, Cambridge, UK, catalog ab34710, 1:800), collagen III (Abcam, catalog ab7778, 1:800), TGF-β1 (Cell Signaling Technology, catalog 3711, 1:1000), p-smad2 (Cell Signaling Technology, catalog 3108, 1:1000), smad2 (Cell Signaling Technology, catalog 5339,1:800), p-smad3 (Cell Signaling Technology, catalog 9520, 1:1000), smad3 (Cell Signaling Technology, catalog 9523, 1:1000), and GAPDH (ZSGB-BIO, Beijing, China, catalog TA-08, 1:1000) at 4 °C overnight followed by the secondary antibody for 1 h. GAPDH was used as internal control. The bands were pictured using GelDox XR System (Bio-Rad, CA, USA). Quantity One software was used to quantify the intensity of the bands.

### Immunofluorescence staining

Fibroblasts were treated with 4% buffered paraformaldehyde for 20 min at room temperature. Then, 1% BSA and 0.1% Triton-X were used to block the membrane for 2 h at room temperature and then treated with the primary antibodies against NLRP3, caspase-1, IL-1β, collagen I, collagen III and TGF-β1 (1:200) at 4 °C overnight before treatment with the secondary antibody for 1 h at room temperature. The nuclei were stained with DAPI (Beyotime, Shanghai, China). The results were captured using a fluorescence microscope. The immunostaining results were quantified and statistically analyzed using Image-Pro Plus 6.0.

### Immunohistochemical analysis

Left ventricle samples were fixed in 4% paraformaldehyde and embedded in paraffin. Samples were then cut into 5 μm-thick sections and stained with the primary antibody against NLRP3, caspase-1, IL-1β and GSDMD-N (1:200) at 4 °C overnight, followed by the secondary antibody. Then, the sections were stained with diaminobenzidine and images were captured by a fluorescence microscope (Nikon 80i, Otawara, Tochigi, Japan).

### Luciferase assay

Both KCNQ1OT1 and caspase-1 3′-UTRs contain conserved miR-214-3p-binding sites. The mutated 3′-UTRs of KCNQ1OT1 were synthesized by RIBOBIO (Guangzhou, China), and amplified by PCR. The PCR fragment was cloned into the XhoI and NotI sites downstream of the luciferase gene in the psi-CHECK2 vector. The 3′-UTR luciferase vector was co-transfected with miR-214-3p mimics into HEK 293 cells using Lipofectamine 200 (Invitrogen), and Renilla luciferase reporters were used as internal controls. A luciferase activity assay was performed after 48 h using the Dual-Luciferase Reporter Assay System (Promega Biotech Co., Ltd.) according to the manufacturer′s instructions. Similarly, wild-type and mutated type CASP1 3′-UTR were synthesized for use to detect the relationship between CASP1 and miR-214-3p.

### Data analysis

Values were analyzed with Graphpad Prism 6 and were presented as the mean ± SD. Unpaired student’s *t* tests were performed to compare the differences between the two groups. One-way analysis of variance combined with Bonferroni posttest were performed to compare the differences between the two or different groups. *P* < 0.05 was considered statistically significant.

## Electronic supplementary material

Supplementary Figure legend-clean

Supplementary Figure 1

Supplementary Figure 2
