# Supplementary material for: Silencing long non-coding RNA Kcnq1ot1 alleviates pyroptosis and fibrosis in diabetic cardiomyopathy
Source: Cell Death Dis. 2018 Sep 24;9(10):1000. doi: 10.1038/s41419-018-1029-4 (PMC6155223; doi:10.1038/s41419-018-1029-4)
Supplement: Supplementary file 1 — Supplementary Figure legend-clean [file 41419_2018_1029_MOESM1_ESM.docx]

**Supplementary Figure**

**Supplementary Figure 1** Kcnq1ot1 was silenced using different siRNAs in cardiac fibroblasts. (a) The expression levels of Kcnq1ot1 after transfection with si-Kcnq1ot1-1, si-Kcnq1ot1-2 and si-Kcnq1ot1-3 were detected by qRT-PCR. (b) The relative mRNA expression levels of caspase-1 were detected by qRT-PCR in each group. **P*<0.05 versus the control group. *#P*<0.05 compared with the HG group. *n*=3 in each group.

**Supplementary Figure 2** miR-214-3p has binding sites for both Kcnq1ot1 and caspase-1. (a) The binding sites of KCNQ1OT1 on miR-214-3p as well as that for the mutant sequence are shown. (b) Wild-type KCNQ1OT1 (WT) and the mutant type (MUT) were cloned downstream of luciferase vector. KCNQ1OT1 WT and MUT were cotransfected with miR-NC and miR-214-3p mimics into HEK293 cells. Luciferase activity was detected using the luciferase assay. The relative Rluc/Luc ratio is shown. (c) Targetscan bioinformatics predicted the binding sites of caspase-1 and miR-214-3p and the mutant sequence of caspase-1. (d) Wild-type caspase-1 (WT) and mutant (MUT) were cloned downstream of luciferase vector. Caspase-1 WT and MUT were cotransfected with miR-NC, miR-214-3p mimics, AMO-NC and AMO-miR-214-3p into HEK293 cells. Luciferase activity was detected using the luciferase assay. The relative Rluc/Luc ratio is shown. **P*<0.05 between the marked groups.
