# Supplementary figures and images for: Silencing long non-coding RNA Kcnq1ot1 alleviates pyroptosis and fibrosis in diabetic cardiomyopathy
Source: Cell Death Dis. 2018 Sep 24;9(10):1000. doi: 10.1038/s41419-018-1029-4 (PMC6155223; doi:10.1038/s41419-018-1029-4)

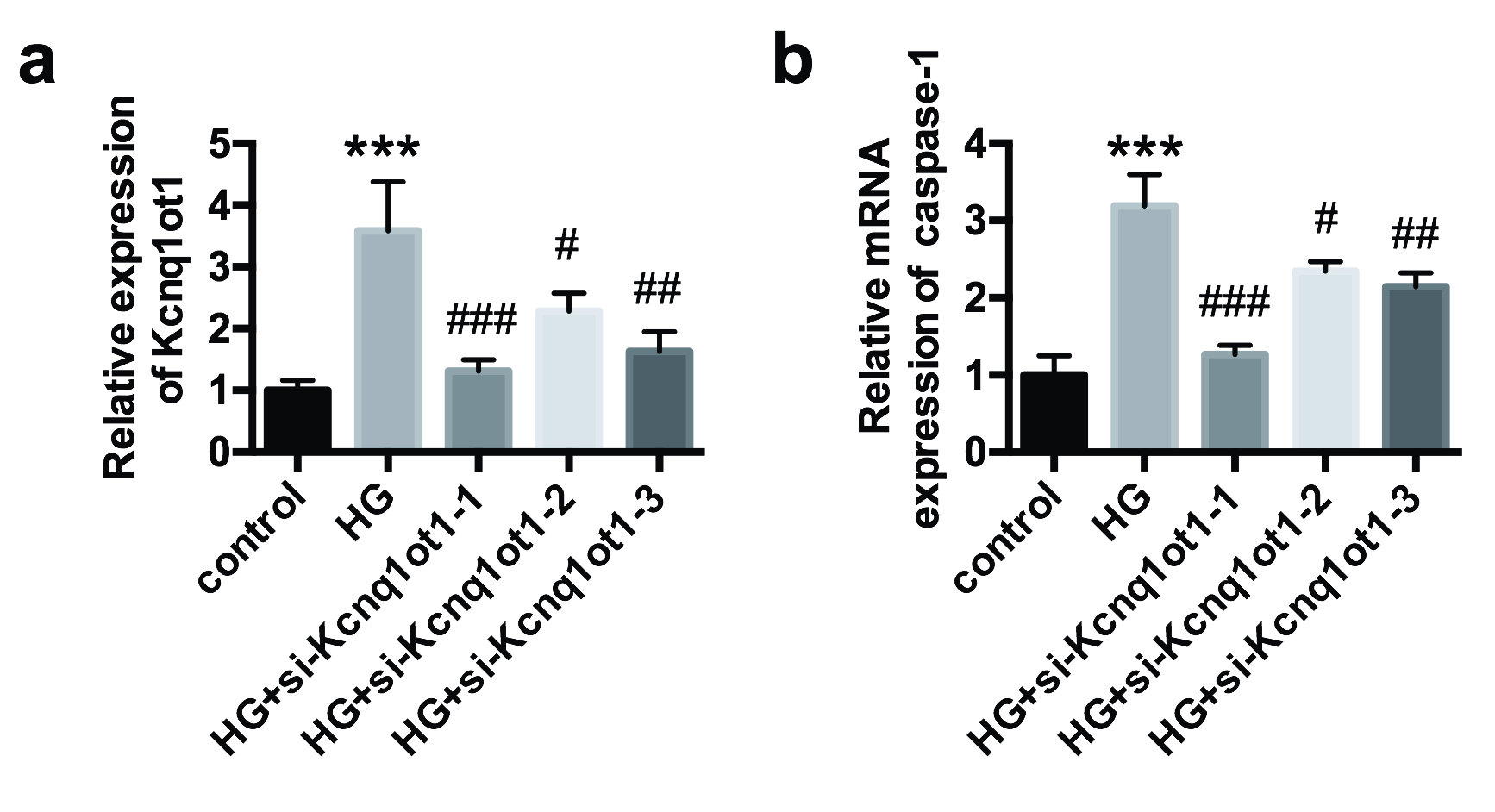

Supplement: Supplementary file 2 — Supplementary Figure 1 [file 41419_2018_1029_MOESM2_ESM.tif]

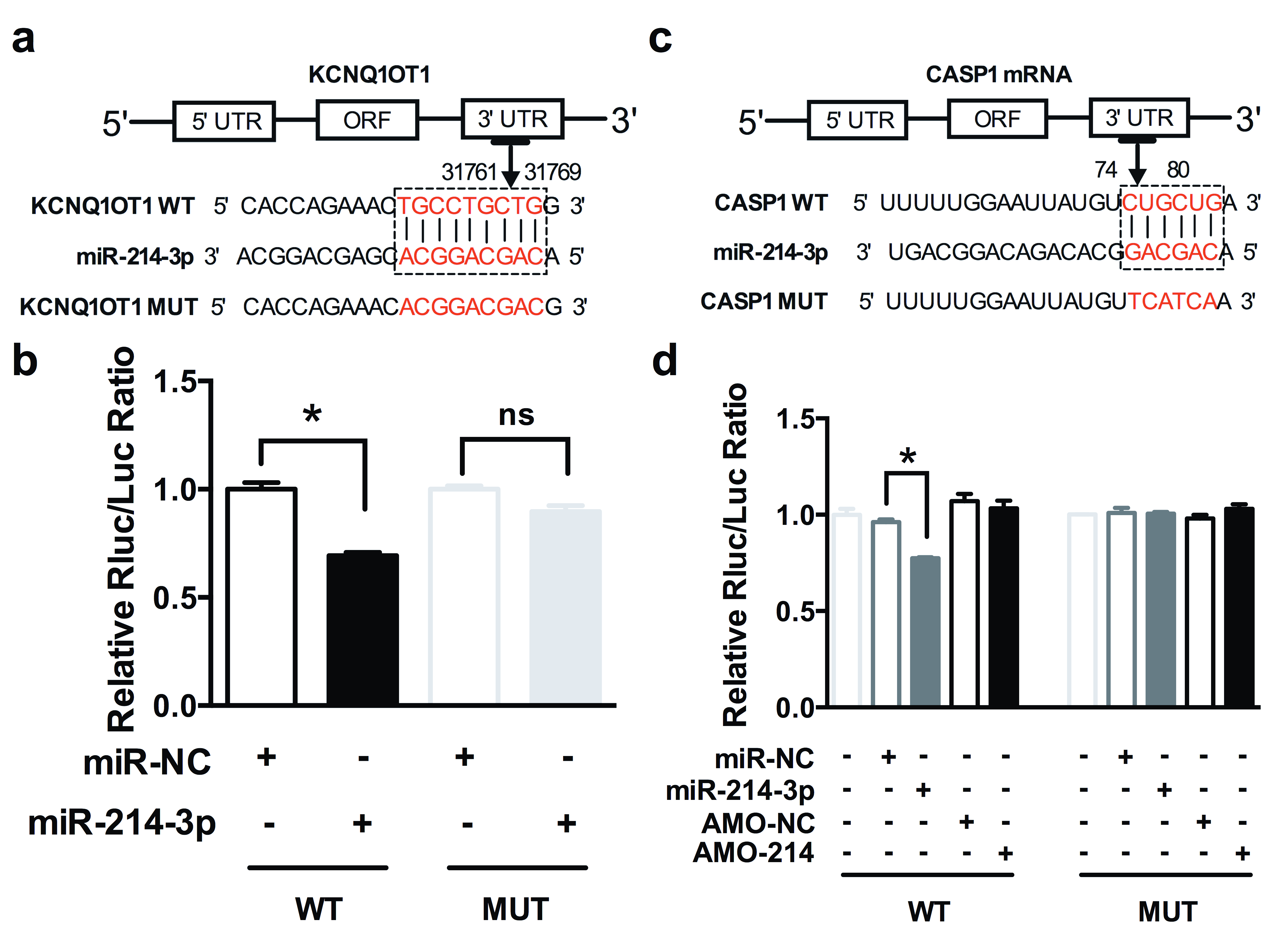

Supplement: Supplementary file 3 — Supplementary Figure 2 [file 41419_2018_1029_MOESM3_ESM.tif]
